# Distribution of H_2_S in the 10103 excavation working face of the Baozigou coal mine

**DOI:** 10.1038/s41598-024-56754-3

**Published:** 2024-03-14

**Authors:** Niujun Jia, Baoshan Jia, Jingxue Yan, Jinyi Zhang

**Affiliations:** 1 College of Safety Science and Engineering, Liaoning Technical University, Fuxin, 123000 Liaoning China; 2 Key Laboratory of Mine Thermal Power Disaster and Prevention, Liaoning Technical University, Ministry of Education, Fuxin, 123000 Liaoning China

**Keywords:** Migration law, Numerical simulation, Excavation working face, H_2_S concentration, Coal mine, Environmental sciences, Engineering

## Abstract

Based on the production conditions of the 10103 excavation working face of the Baozigou coal mine, this paper analyzes the potential sources of H_2_S and the expected emission concentrations of H_2_S in the working face. Considering the previous engineering practice for controlling H_2_S disasters in coal mine working faces, numerical simulations were conducted to investigate air flow and H_2_S migration and diffusion in the tunnel in the excavation working face. The migration and distribution of H_2_S in the coal seam mining face were studied, and the effects of outlet wind speed, duct location, and duct diameter on the H_2_S concentration distribution were explored. The higher the outlet wind speed, the more conducive to the emission of H_2_S gas, but too high a wind speed will be detrimental to the concentrated extraction and purification absorption of H_2_S; the closer the outlet position of the air duct is to the end of the working surface, the lower the H_2_S concentration in the vortex area at the corner; the air duct If the diameter is too small, the harmful gases released from hard-to-break coal cannot be entrained and taken away. When the diameter of the air duct is too large, the entrainment volume during the jet process will be expanded. To verify the field distribution of H_2_S concentration at the bottom, middle, and top of the boring machine, a CD4-type portable H_2_S instrument was used to analyze the distribution of H_2_S near the excavation working face.

## Introduction

Hydrogen sulfide (H_2_S), a colorless, slightly sweet, and highly toxic gas with a rotten egg flavor, is a toxic and harmful gas found in coal mines^1^ that seriously threatens the life, health, and safety of miners^2^. H_2_S-containing oil and gas reservoirs are widely distributed in North America, the Arabian Gulf, Europe, South Asia, southwest and northwest China, and other regions around the world^3^. H_2_S accidents have occurred frequently in recent years. In January 2021, a hydrogen sulphide leak at a chemical plant in India resulted in the hospitalisation of at least seven workers. In June 2021, a hydrogen sulphide leak in the Czech Republic resulted in the deaths of two people. In July 2021, a hydrogen sulphide leak in Japan resulted in seven people being unwell and one person suffering from hydrogen sulphide poisoning. In July 2021, a hydrogen sulphide leak in Japan caused seven people to be unwell and one person to be poisoned by hydrogen sulphide. In recent years, China has had H_2_S accidents in more than 10 provinces, including Xinjiang, Inner Mongolia, Yunnan, Guizhou, and Sichuan. Therefore, it is necessary to treat and control H_2_S. Domestic and foreign experts and scholars have carried out research on the migration of H_2_S in coal mines.

Li Xianfeng^4^ established a prediction system for anomalous H_2_S accumulation in the 5-405 working face of the North Shanxi coal mine, reduced the speed at which mining operations advanced, and improved safety measures to ensure the safety of the working face. Smith et al.^5^ studied and compared the sulfur isotopes of organic sulfur, inorganic sulfur such as pyrite, and H_2_ gas in the Collinsville coal mine and analyzed that the abnormal H_2_S content in the coal seam was caused by the intrusion of sulfur-containing magma during the geological evolution process, and the H_2_S formation in the coal mine was because of sulfate biological reduction and thermochemical decomposition of organic sulfur. Mochizuki^6^ adopted the fate of sulfur during carbonization at 3 °C/min of seven caking coals with carbon and sulfur contents of 80–88 and 0.55–1.8 mass%-daf, respectively, has been studied using a flow-type fixed-bed quartz reactor to examine its effect on coal fluidity in caking coal. Wang Wei et al.^7^ proposed the use of an air curtain fan to close the shaft and prevent H_2_S overflow. Based on potential flow superposition and the theory of fluid mechanics, a mathematical model of the flow field of the air curtain fan under spatially confined conditions was established. Wang Fuzhong^8^ used a portable CD4-type H_2_S instrument to detect the volume fraction of H_2_S gas and studied the mechanisms of H_2_S extraction and purification. Hui Yongfu^9^ used the Fick diffusion theorem to describe the migration of H_2_S in the excavation working face; considering a gas flow model of the excavation working face constructed using the Navier–Stokes equations, a physical model for H_2_S migration and diffusion in the excavation working face was established. Huang Lining^10^ proposed the pre-injection of H_2_S absorption liquid into the working face using water injection technology based on segmentation into three pressure zones (pressure relief, stress concentration, and original stress zones) and the high-pressure injection of H_2_S absorption liquid into the cutting head. Asaoka^11^ found that in coal seams with a high oxidation degree, H_2_S will be adsorbed on fly ash and a REDOX reaction will occur. Most H_2_S will be oxidized to elemental sulfur, and a small part will be oxidized to sulfate.Gao^12^ obtained coal samples from the Shanxi Shaping coal mine to investigate the occurrence of H_2_S in low-sulfur coal seams. The adsorption mechanism of H_2_S on coal was explored, and an equation for H_2_S adsorption in coal seams was derived based on adsorption experiments.

Therefore, studying the diffusion laws of underground hydrogen sulfide and finding ways to reduce the concentration of underground H_2_S are of great significance to corporate safety and social security. Based on the production conditions of the 10103 excavation working face of the Baozigou coal mine and previous engineering practices for controlling H_2_S disasters in coal mines, this paper explores the migration and distribution of H_2_S in a coal mining face based on numerical simulation. The effects of outlet wind speed, duct location, and duct diameter on the H_2_S concentration distribution were studied, and the distribution of H_2_S near the excavation working face was analyzed on site. Provide certain theoretical support and improvement suggestions to optimize the working conditions of the mine excavation working face, reduce the H_2_S concentration, and protect the workers.

## Overview of the Baozigou coal mine

### General description of the coal seam and coal quality in the Baozigou coal mine

The Baozigou coal industry is located in Shanxi Province. The coal seams that can be mined are the No. 8 coal seam, No. 9 coal seam, and No. 10 + 11 coal seam. The No. 9 and No. 10 + 11 coal seams contain coking coal and fertilizer coal, and the formation inclination is generally less than 25°. According to the Code for Geological Exploration of Coal and Peat (DZ/T0215-2002)^13^, the estimated coal resource characteristics are as follows: minimum coal seam mining thickness, 0.70 m; maximum ash content (Ad), 40%; and maximum sulfur content (St, d), 3%. This paper mainly focuses on the No. 9 and No. 10 + 11 coal seams. The chemical and process properties of this coal, as shown in Table 1.

**Table 1:** Chemical properties and technological properties of coal.

Name	Raw coal (%)	Average (%)	Floating coal (%)	Average (%)	Coal sample characteristics
No. 9 coal Seam ash (Ad)	9.87–25.17	18.34	4.32–12.80	8.52	**Extra low ash—high ash, high sulfur, high calorific value—extra high calorific value, strong bond—extra strong bond coking coal and fat coal**
No. 9 coal seam: sulfur content (St, d)	2.52–4.62	2.90	2.24–3.21	2.69	
No. 10 + 11 coal seam: ash (Ad)	17.05–31.28	21.68	6.99–14.93	9.92	Coking coal and fat coal with low ash—high ash, high sulfur, low calorific value—high calorific value, strong bond—especially strong bond
No. 10 + 11 coal seam: sulfur content (St, d)	2.21–3.64	2.95	1.98–4.07	2.95	

### Origin of H_2_S in the coal seam of the Baozigou mine

#### Analysis of H_2_S genetic type

A previous study on the origin of H_2_S in coal seams^14^ revealed five main sources of H_2_S: thermochemical decomposition, sulfate chemical processes, bacterial sulfate reduction (BSR), biodegradation, and magmatic processes. BSR and biodegradation are biochemical sources, whereas thermochemical decomposition and sulfate chemical processes are thermochemical sources.

#### Analysis of H_2_S genetic type in the coal seam of the Baozigou mine

Numerous pyrite, ferrous debris, and Ordovician gypsum strata are found in the 9#, 10#, and 11# coal-bearing strata of the mine. Thus, the formation of H_2_S in the coal seam of the Baozigou mine can be attributed to thermochemical sulfate reduction (TSR) and BSR. The H_2_S genetic type analysis of the No. 9# coal seam BSR is shown in Table 2.

**Table 2:** H_2_S genetic type analysis of the No. 9# coal seam BSR.

Name	9# coal seam gas components	δ13CO_2_ value in coalbed methane	δ13CO_2_ values in regional water bodies	Methane carbon isotope value
Composition/Value	CH_4_, N_2_, CO_2_	− 11.5–18.1‰	− 11.2–18.1‰	< − 50‰
Name	δ34 value in coal mine groundwater	Average δ34 value of pyrite in coal	Average δ34 value of H_2_S gas in coal seam	∆δ34 total
Composition/Value	− 0.6‰	10.2‰	− 12‰	> 22%

1. Causes of BSR: Coal seams 9# and 10 + 11# in the Baozigou coal mine are located in the Shanxi Formation of the Lower Permian Series and the Taiyuan Formation of the Upper Carboniferous Series, which are terrestrial and marine coal-bearing structures. Coal seams 9# and 10# are located in the tidal flat environment of the lagoon and are affected by seawater erosion, resulting in a large amount of sulfur accumulation in the coal. H_2_S gas is then formed during the accumulation of sulfur. Based on measurements of coal vitrinite reflectance and fluid inclusion temperature, the maximum vitrinite reflectance of coal from seam 9# is between 0.51% and 0.75%, and the maximum temperature during coal formation and burial does not exceed 120 °C. These temperature conditions are appropriate for BSR. Moreover, the H_2_S content of the main coal seam in most mines in this region is very high, and a large amount of berberium pyrite, a by-product of BSR, is distributed in the coal seam. The distribution of H_2_S can be divided into several zones based on its concentration, which is in line with typical BSR formation characteristics. According to the sedimentary environment of the coal seam, pyrite morphology, fluid inclusion temperature, vitrinite reflectance, thermal evolution history, sulfur isotope characteristics, and hydrological characteristics, we can conclude that BSR is the main origin of H_2_S gas in the coal seams of the Baozigou mine.
2. Sulfate TSR. The TSR chemical reaction proceeds as follows:
  1 $${\text{4CH}}_{{2}} + {\text{3SO}}_{{4}}^{{ - {2}}} + {\text{2H}}_{{2}} {\text{O }} \to {\text{4CH}}_{{2}} + {\text{3H}}_{{2}} {\text{S}} + {\text{6OH}}^{ - }$$
  2 $${\text{CH}}_{{4}} + {\text{CaSO}}_{{4}} \to {\text{CaSO3}} + {\text{H}}_{{2}} {\text{S}} + {\text{2H}}_{{2}} {\text{O}}$$
  3 $${\text{C}}_{{2}} {\text{H}}_{{6}} + {\text{2CaSO}}_{{4}} \to {\text{2CaSO}}_{{3}} + {\text{H}}_{{2}} {\text{S}} + {\text{S}} + {\text{2H}}_{{2}} {\text{O}}$$

As indicated in the above chemical reaction formulae, the essence of the TSR reaction is the reaction between hydrocarbons and sulfate. The chemical reaction requires calcium sulfate solution rather than solid gypsum mineral; thus, the reaction must involve mine water. Mine water is abundant in the Baozigou mine. Coal seams 9#, 10#, and 11# are karst fissure water-filled deposits dominated by roof water intake, and the roof aquifer of the coal seam is a limestone aquifer with a K1–K7 sandstone bottom. The upper part of the Carboniferous system is mainly composed of mudstone or limestone with developed karst fractures, providing a location for TSR. Based on the geologic characteristics of the Baozigou mine, the coal-bearing strata in the mining area contain a large amount of gypsum ore. The Upper Carboniferous Taiyuan Formation is mainly composed of gray-light gray bangle and thick gypsum, gray-black mudstone, sandy mudstone, gray medium fine-grained sandstone, 3–4 layers of limestone, and 3–8 layers of coal with an average thickness of 85.05 m and is integrated with the lower strata. Moreover, in the process of coal formation, coal cracking under the action of high temperature and high pressure produces large amounts of hydrocarbon gases such as methane and ethylene, which provides sufficient reducing agent for TSR. Thus, it can be inferred that a large amount of H_2_S gas is present in the coal seam contact of the Taiyuan Formation aquifer of the Upper Carboniferous System and the Shanxi Formation of the Lower Permian system.

## H_2_S migration in the 10103 excavation working face

### Analysis of air flow characteristics in the excavation working face

**Figure 1:**
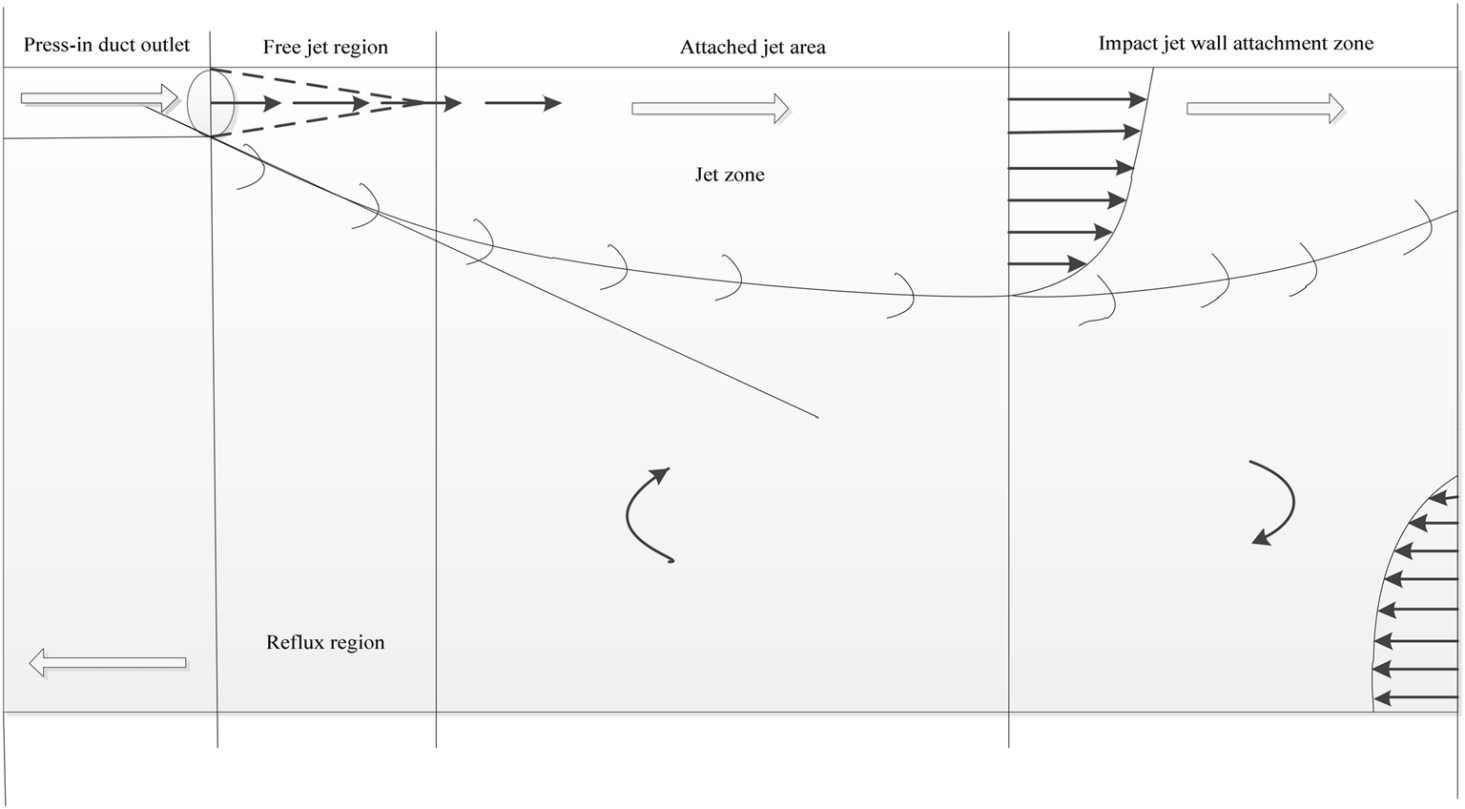
Distribution of air flow in the excavation working face.

The excavation working faces of coal mines in China are widely subjected to pressurized ventilation^15^, and the air duct is hung on one side wall of the tunnel in it. After fresh air flows out of the air duct, a limited wall attachment jet is formed at its end piece, which is closed due to the restriction of the side wall of the tunnel, as shown in Fig. 1.

As shown in Fig. 1, fresh air flows forward along the side of the tunnel in the form of a free jet after it is emitted from the duct. The velocity of air flow in this area is consistent with that at the exit of the duct. As the air continues to flow forward, the diameter and flow rate of the jet increase gradually during the flow process. Due to the obstruction of the tunneltunnel wall, the jet cannot take in air on one side of the wall, but it can continuously take in air on the other side of the wall and expand outward, thus forming a jet area attached to the wall. When the airflow approaches the end of the working face, the velocity of the airflow is greatly reduced, the airflow is blocked from moving further in that direction, and the jet area attached to the wall is forced to form under the pushing action of the airflow in the jet zone. Due to the continuity of air flow, the air flow in the impact jet wall attached area deflects and flows outward along the other side wall of the tunnel, forming a backflow area. Because the flow direction in the jet zone is opposite to that in the return zone, the jet constantly enrolls the external air. Therefore, the size and intensity of the vortex region are closely related to the wind speed at the outlet of the wind duct and the installation position of the wind duct.

### Numerical simulation of H_2_S migration and distribution in the excavating face

#### Basic assumptions and construction of the model

**Figure 2:**
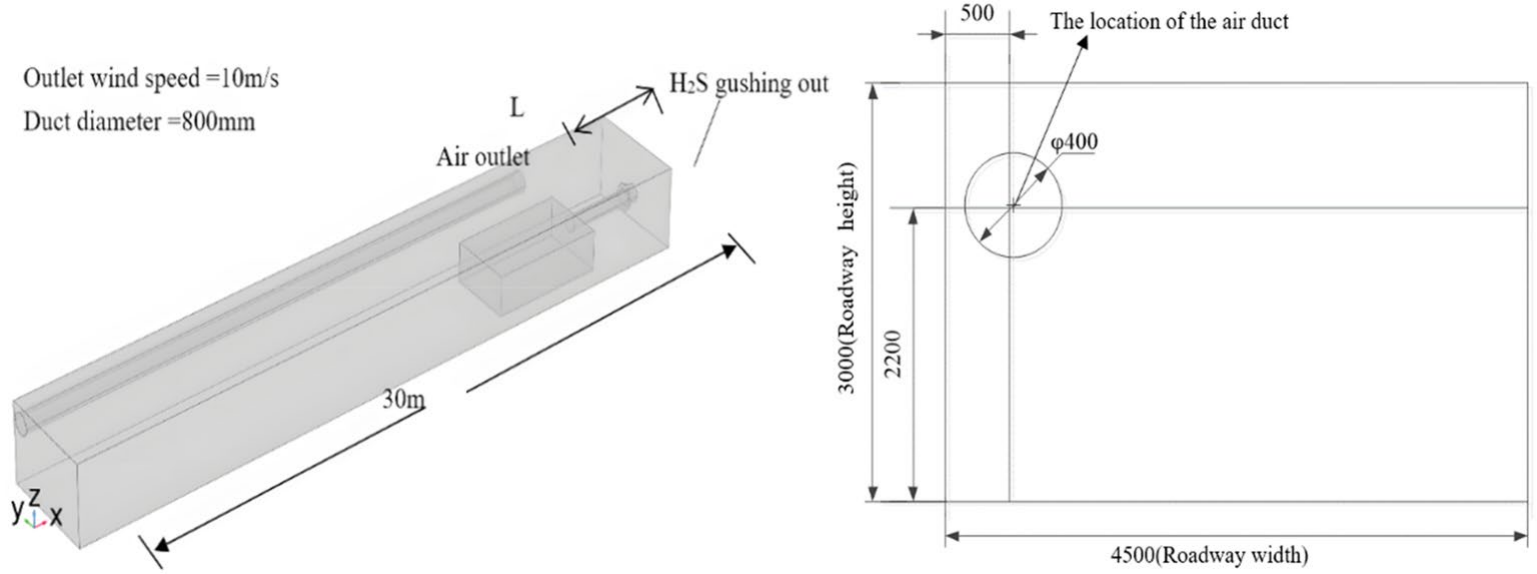
Numerical simulation model(mm).

Numerical simulations were carried out based on the 10103 excavation working face of the Baozigou mine of Shan Coal Group. The tunnel section of the working face is rectangular, the tunnel width is 4.5 m, the tunnel height is 3 m, the section area is 13.5 m^2^, the diameter of the air duct is 0.8 m, the center line is 2.4 m from the floor of the tunnel, and the air outlet of the windpipe is 5 m from the face head. The boring machine body was simplified as a rectangle with dimensions of 5 m × 3 m × 1.48 m. The distance between the boring machine body and the working face was 4.5 m. A distance of 30 m from the working face head was selected to establish a simplified physical model, which is shown in Fig. 2.

#### Establishment of the mathematical model

1. Air flow model of the tunnel in the excavation working face. In this paper, the three-dimensional steady-state incompressible Navier–Stokes equation was adopted as the governing equation for the flow field of the entire tunnel^16^. The turbulence model was based on the eddy viscous model, and turbulent flow was based on a *k*–*ε* model. This model only considers momentum transfer and ignores the influence of heat transfer, and the equation only solves for the dependent variable *u* and pressure *p*. The specific form is as follows^17,18^:
  Continuity equation:
  4 $$\rho \nabla \cdot \left( u \right){ = 0}$$
  Equation of motion:
  5 $$\rho \frac{\partial u}{{\partial t}} + \rho \left( {u \cdot \nabla } \right)u = \nabla \left[ { - pl + \left( {\mu + \mu_{T} } \right)(\nabla u + \left( {\nabla u)^{T} } \right)} \right] + F + \rho g$$
  Turbulent kinetic energy k equation:
  6 $$\rho \frac{\partial k}{{\partial t}} + \rho \left( {u \cdot \nabla } \right)k = \nabla \cdot \left[ {\left( {\mu + \frac{{\mu_{T} }}{{\sigma_{K} }}} \right)\nabla_{K} } \right] + P_{K} - \rho \varepsilon$$
  Dissipation rate ε equation:
  7 $$\rho \frac{\partial \varepsilon }{{\partial t}} + \rho \left( {u \cdot \nabla } \right)\varepsilon = \nabla \cdot \left[ {\left( {\mu + \frac{{\mu_{T} }}{{\sigma_{K} }}} \right)\nabla \varepsilon } \right] + C_{\varepsilon 1} \frac{\varepsilon }{k}P_{K} - C_{\varepsilon 2} \rho \frac{{\varepsilon^{2} }}{k}$$
  in which
  8 $$\varepsilon = ep$$
  9 $$\mu_{T} = \rho C_{\mu } \frac{{k^{2} }}{\varepsilon }$$
  10 $$P_{k} = \mu_{T} \left[ {\nabla u \cdot \left( {\nabla u + \left( {\nabla u} \right)^{T} } \right)} \right]$$
  where $\nabla$ is a Hamiltonian operator; *u* is speed in m/s; ρ is the mixture density in kg/m^3^; *p* is the pressure in Pa; *l* is the turbulence length; *µ* is the viscosity coefficient of laminar flow in Pa∙s; *ε* is the dissipation rate of turbulent kinetic energy in m^2^/s^2^; *µ*_*T*_ is the turbulence viscosity coefficient in Pa s; *C*_*ε1*_, *C*_*ε2*_, *C*_*µ*_, and σ_*k*_ are empirical constants with values of 1.42, 1.68, 0.09, and 1, respectively; *g* is the acceleration of gravity (9.81 m/s^2^); and *P*_*k*_ is the rate of change in kinetic energy caused by the change in shear force.
2. H_2_S migration and diffusion model. The migration of H_2_S in the stope follows the law of hydrodynamic dispersion. Because the concentration of H_2_S is much lower than its flux concentration, the Fickian method can be used to describe the diffusion term in material transport. The control equation of H_2_S migration in stope is obtained according to the mass conservation law and the convection diffusion equation:
  11 $$\frac{{\partial c_{i} }}{\partial t} = \nabla \cdot D_{i} \nabla c_{i} - \nabla \cdot uc_{i} + R_{i}$$
  12 $$\rho = c_{i} V_{L} \cdot 10^{ - 3} \rho_{H} + \left( {1 - c_{i} \cdot V_{L} \cdot 10^{ - 3} } \right) \cdot \rho_{a}$$
  13 $$N_{i} = - D_{i} \nabla c_{i} + uc_{i}$$
  where C_*i*_ is the dissolved concentration, mol/m^3^; *D*_*i*_ is the diffusion coefficient of H_2_S in air in m^2^ S; *R*_*i*_ is the source term (i.e., the increase in H_2_S per unit time per unit volume in mol/m^3^·s); *u* is the average flow rate in m/s; *ρ*_*H*_ and *ρ*_*a*_ are the density of H_2_S and air in kg/m^3^, respectively; and *V*_*L*_ is the volume of the gas at the standard condition (22.4 L/mol).

Because H_2_S is a solute, the velocity and pressure fields can be calculated by the turbulence model for diffusion Eqs. (4–7) and reach a new equilibrium state after a period of time. This is a multi-physical-field process in which solute diffusion and fluid flow are coupled. By solving Eqs. (4–9), the migration and diffusion laws of H_2_S in the mine are obtained along with the distribution characteristics of H_2_S concentration in the tunnel and working face.

#### Settings of solution conditions and model calculation parameters

Two counter-rotating axial-flow fans were adopted at the 10103 working face of the Baozigou coal mine. The air supply volume was 300 m^3^/min, the wind speed at the outlet of the blower was approximately 10 m/s, the wind speed at the outlet of the tunnel was 0.62 m/s, the turbulence intensity was 3.33%, and the hydraulic diameter was 3.2 m. The other parameters calculated based on the real field data are shown in Table 3.

**Table 3:** Model calculation parameters.

Physical quantity	Parameter	Parameter value	Unit
C_0_	Inflow of H_2_S	5.38 × 10^−3^	mol/m^3^
*v*	Outlet wind speed	10	m/s
µ	Dynamic viscosity of mixture	17.9 × 10^−6^	Pa s
P_0_	Initial tunnel pressure	0.101	MPa
$\rho_{H}$	The density of H_2_S	1.593	kg/m^3^
$\rho_{a}$	Tunnel air density	1.29	kg/m^3^
D_i_	Diffusion coefficient of H_2_S in air	1.58 × 10^−5^	m^2^/s
V_H_	Inflow velocity of H_2_S	8.3 × 10^−4^	m/s

### Analysis of H_2_S migration in the excavating face

#### Flow field analysis of the excavation working face

**Figure 3:**
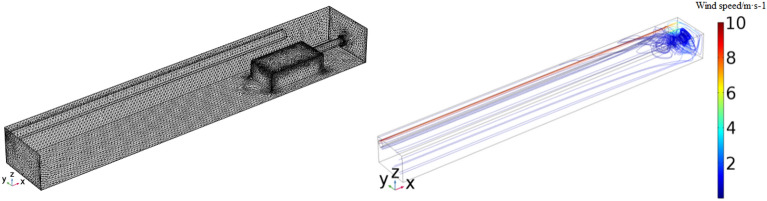
Model grid division and velocity flow diagram of the tunnel in the excavation working face.

In this model, in order to improve the computational precision of the gas outflow border, set the boundary grid as a free-separated triangular grid; the grid between the grinding machine and the border interface, alongside the grading machine, has a larger gradient of positioning of parts where the corner finishing process is carried out; the alley and windscreen parts are freely separated by the quadrilateral grid processing. The model is divided into 35,864 grids. The grid diagram after division and the velocity flow diagram of the tunnel in the excavation working face are shown in Fig. 3.

**Figure 4:**
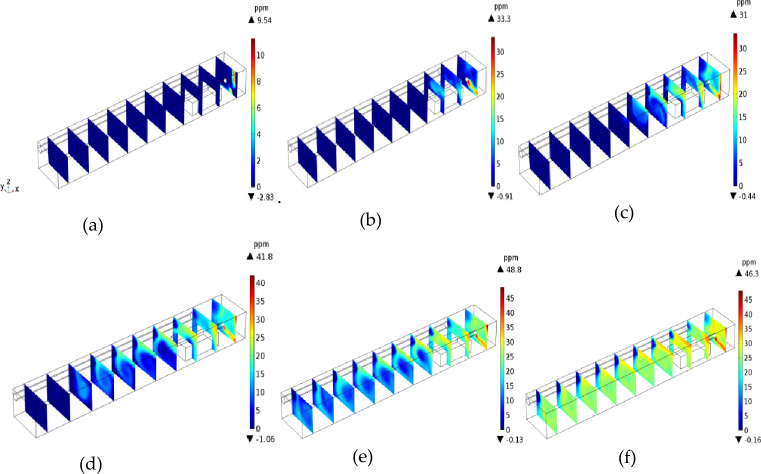
Migration and diffusion of H_2_S.

As shown in Fig. 4, the high-speed air flow leaves the duct and backflows outward along the excavation tunnel. The vortex formed by the low-speed air flow is formed within 4 m of the rear of the working face. The upper wind with high wind speed flows out in two ways after being reflected by the return air corner. In the process of jet flow, the flow line is further closed as the wind speed decreases, and the vortex forms again near the left side wall of the tunnel within 6–10 m from the working face. Another part of the return air flows back along the right side wall of the tunnel. Meanwhile, the flow line in the middle of the tunnel is sparsely distributed; thus, the air volume is relatively small. The return air flow of the tunnel approximates laminar flow.

#### Analysis of H_2_S migration in the excavation working face

To study the migration of H_2_S after it exits the head, the flow field and concentration field near the excavation working face were calculated in a transient way. To clearly display the calculation results, 10 reference planes were uniformly selected in the direction of the tunnel (positive direction of the X-axis) with an average surface spacing of 2.7 m. The calculation results are shown in Fig. 4.

Assuming the continuous operation of the tunnel boring machine (TBM), the simulation results indicate that H_2_S diffusion reached a steady state after 118 s. As shown in Fig. 4, H2S is most likely to gather in the lower corner of the return air side of the TBM and around the TBM at 6–10 m away from the TBM under the U-type ventilation system of the TBM working face. H_2_S can diffuse outward from the working face in three stages: gravity expansion, passive expansion, and uniform expansion.

1. *Gravity diffusion stage* As shown in Fig. 4a,b, in the early stage of H_2_S diffusion, the H_2_S concentration is relatively high in the lower corner of the tunnel floor and the return air side of the working face. At this stage, H_2_S emitted from the mining process has not had time to mix with the air. H_2_S emitted from the excavation working face accumulates in the lower corner of the return air of the working face, where the wind speed is low due to the settlement of the wall air flow and gravity.
2. *Passive diffusion stage* As shown in Fig. 4c,d, the H_2_S in the lower corner gradually diffused to the roof and left side of the tunnel, and the H_2_S concentration near the TBM driver (6–10 m away from the working face) increased rapidly. At this stage, most of the high-concentration H_2_S in the lower corner flowed with the wind to the jet above the inclined side of the tunnel. As the wind speed decreased, the H_2_S mixed with air and its concentration thinned, and the H_2_S concentration at the roof and left wall of the tunnel also increased.
3. *Uniform diffusion stage* As shown in Fig. 4e,f, as the distance from the working face increased, the H_2_S attached to the left side of the tunnel diffused outward in an inverted triangle pattern. Meanwhile, the H_2_S in the lower corner of the right side of the tunnel migrated out along the floor of the tunnel with the wind flow. At this stage, the flow field tended to be stable, and the H_2_S at the roof settled under the action of gravity and gradually diffused evenly with the increase in the distance from the tunnel.

**Figure 5:**
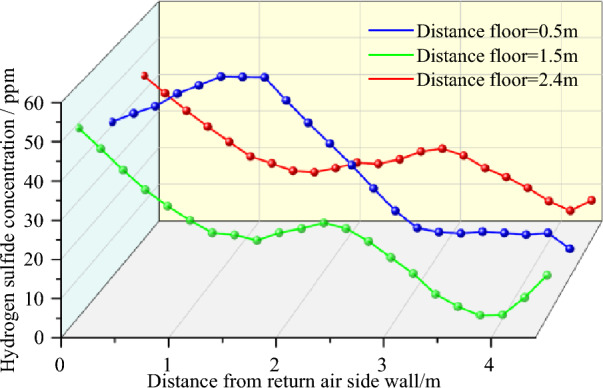
Distribution of H_2_S concentration on the working face.

As shown in Fig. 5, the H_2_S concentration increased moving from the inlet air side to the return air side, and the concentration on the reference line 0.5 m away from the base plate was higher than those at 1.5 and 2.4 m away from the base plate. These results indicate the gravitational diffusion characteristics of H_2_S. At a distance of 0.5 m from the bottom plate, the H_2_S concentration increased in the range of 0–1.7 m from the tunnel wall of the return air side and reached a peak of more than 55 ppm in the range of 1.3–1.7 m from the tunnel wall of the return air side. Due to the transition from gravitational diffusion to passive expansion occurring near this range, the high concentration of H_2_S in the lower corner continued to spread to the top of the TBM with the wind flow; this range should be a priority for reducing H_2_S concentration and for making other improvements. Comparing the concentration curves of the reference lines 1.5 and 2.4 m away from the floor, we found that the two curves were basically the same within 2.7 m from the wall of the return air lane. The H_2_S concentration gradually decreased as the distance from the wall of the return air lane increased; however, the H_2_S concentration on the reference line 2.4 m away from the floor was slightly higher than that on the 1.5-m reference plane. This is because in the passive diffusion stage, part of H_2_S was swept to the top of the tunnel with the wind flow, causing the concentration to increase. After the flow field became relatively stable over 2.7 m, gravity settlement was restored, and the concentration at the 2.4-m reference plane was lower than that of the 1.5-m reference plane. We can conclude that the distribution of H_2_S near the working face is jointly determined by gravity and the flow field. The concentration of H_2_S was high within 2.3 m of the return air side, and the minimum value exceeded 25 ppm. After 2.3 m, the concentration showed a decreasing trend. Therefore, the control of H_2_S within 2.3 m of the return air side from the tunnel wall should be strengthened.

### Effect of outlet wind speed on the H_2_S distribution

The influence of wind speed in the air duct (6, 8, 10, and 15 m/s) on the distribution of H_2_S concentration was investigated. The diameter of the air duct was 0.8 m, and the distance between the outlet of the air duct and the working face was 5 m. The coal wall facing the air duct outlet (i.e., the upper right corner of the tunnel) with dimensions of 800 mm × 800 mm was selected as the H_2_S emission source, and the maximum H_2_S emission rate was 1/34·9 mol/m^2^·s when the TBM cut the top coal. Taking the breathing plane *z* = 1.5 m (i.e., the average height at which workers breathe) as a reference, the *xy* contour cloud map of H_2_S concentration was obtained.

**Figure 6:**
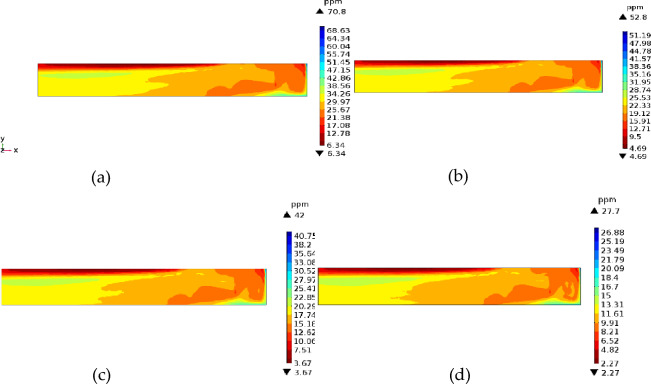
The *xy* cross-section (*z* = 1.5) of the cloud map of H_2_S concentration for different wind speeds.

As shown in Fig. 6, increasing the wind speed had little effect on H_2_S concentration. Upon increasing the wind speed, the zone of high H_2_S concentration moves to the rear area of the tunnel. Due to the increase in wind speed and air volume, the H_2_S concentration of the cross section becomes smaller. For wind speeds of 6, 8, 10, and 15 m/s, the highest concentrations of H_2_S on the return air side of the working face were 70.8, 50.8, 42, and 27.7 ppm, respectively, whereas the lowest concentrations on the inlet air side were 6.34, 4.39, 3.67, and 2.27 ppm, respectively, indicating that high wind speed and large air volume were conducive to the dilution of H_2_S. Therefore, when mining high-sulfur coal mines, the wind speed at the outlet of the duct could be increased to prevent H_2_S accumulation and improve the working environment near the inlet wind side. However, the outlet wind speed should not be too large. With increasing wind speed, H_2_S quickly moves toward the back of the tunnel, and high concentrations of H_2_S will spread to the entire tunnel space in a short time. Therefore, excessive wind speed is not conducive to the extraction and purification of H_2_S, and the optimal wind speed at the outlet of the duct is generally considered to be 10 m/s.

**Figure 7:**
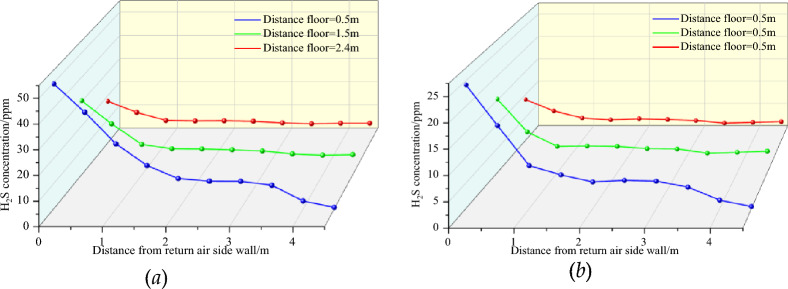
Distribution of H_2_S concentration near the head.

Figure 7 shows the distribution of H_2_S concentration near the working face (*x* = 28, *yz* section) at wind speeds of 8 and 15 m/s. When the wind speed increased from 8 to 15 m/s, the H_2_S concentration on the inlet and return wind side decreased significantly, with a maximum decrease of 45.4%. In addition, during the cutting of top coal by the TBM, the H_2_S concentration does not change much beyond 1 m from the wall of the return air tunnel.

### Simulation of H_2_S distribution under different duct positions

#### Analysis of the air flow field for different duct positions

**Figure 8:**
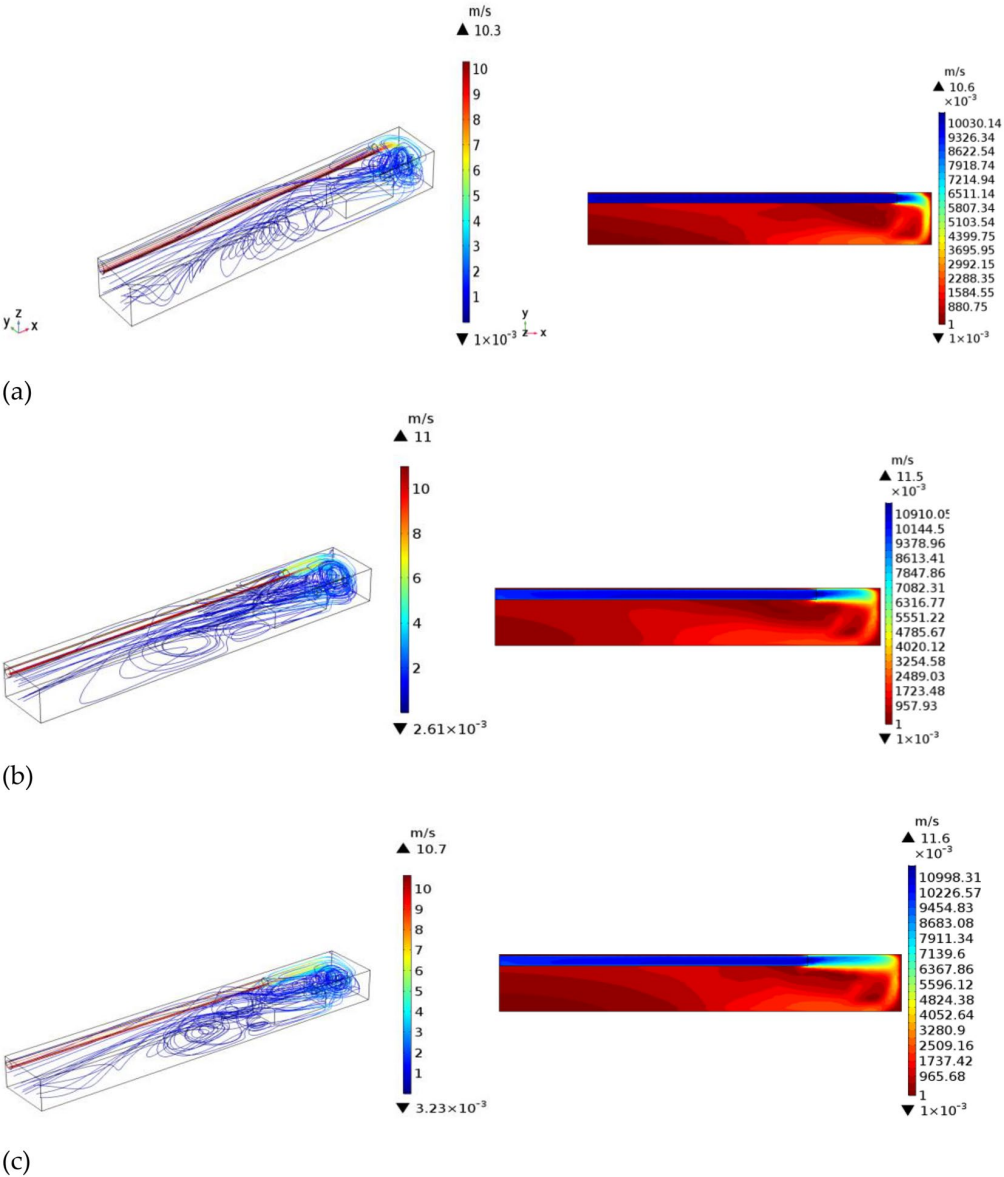
Velocity flow diagrams and contour maps at different duct positions.

To investigate the influence of duct outlet position on the distribution of H_2_S concentration, the distance from the duct to the head was varied (*L* = 3, 5, and 7 m). The outlet wind speed of the air duct was 10 m/s, the diameter of the air duct was 0.4 m, and the air supply temperature was 20 °C. H_2_S gouges from the heading of the tunnel in the excavation working face, and the amount of H_2_S outflow was 1/34·9 mol/m^3^. Figure 8 shows the simulation results.

1. As shown in Fig. 8, for *L* = 3 m, due to the large deflection speed of the near air flow, the vortex intensity was large, and the high outlet wind speed led to a small jet enfranchisement air volume, small rotating air volume, and small vortex area; thus, no vortex in the true sense was formed. After the air flow bounced through the return air corner, it moved rapidly to the rear along the opposite side of the tunnel wall and through the upper part of the TBM.
2. For *L* = 5 m, the airflow deflecting speed was moderate, and the jet entrainment air volume and whirl air volume increased compared with those in the case of *L* = 3 m; as a result, the area of the vortex region increased, but the vortex intensity decreased, and the regional boundary approximately reached the outlet position. As shown in the velocity flow diagrams, the flow line at the top of the TBM was denser at *L* = 5 m than at *L* = 3 m; thus, the air volume increased at *L* = 5 m due to the expansion of the swirling air volume at the vortex boundary and the decreased wind speed.
3. For *L* = 7 m, when the jet reached the end of the working face and was deflected, both the wind speed and vortex strength were small, while the volume of the enrolling air and the rotating air of the jet was large. Compared with *L* = 5 m, the vortex region was larger, but its core region was smaller. Because the distance from *L* = 7 to the end of the working face was relatively small, the wind speed decreased, the flow line distribution of the upper part of the tunnel was relatively uniform, and the return air slowly diffused to the side wall of the tunnel.

Therefore, within the effective range of the jet, when the air outlet speed was 10 m/s, the vortex region formed on the opposite side of the wind duct increased in area as the distance between the wind duct outlet and the end of the working face increased. However, the area of the core vortex region first increased and then decreased with *L*, and the area of the core region was maximized at *L* = 5 m.

#### Effect of duct locations on the H_2_S concentration distribution

**Figure 9:**
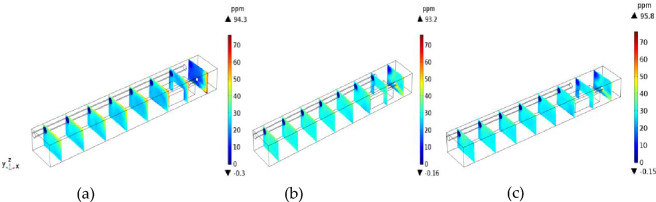
The *yz* cross-sections of H_2_S concentration.

**Figure 10:**
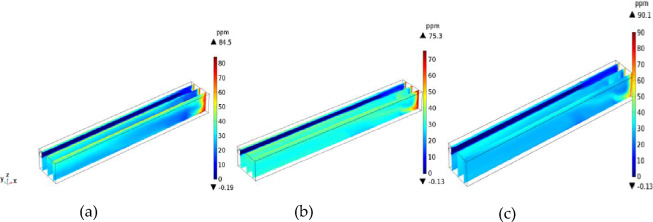
The *xz* cross-sections of H_2_S concentration.

**Figure 11:**
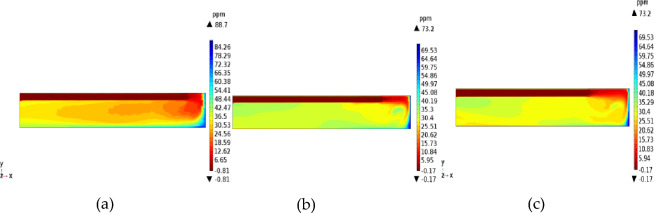
Distributions of H_2_S concentration at different distances from the duct outlet to the working face.

**Figure 12:**
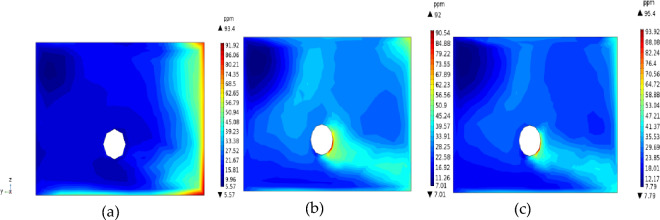
Contour map of H_2_S concentration at *x* = 28 m.

The distance from the duct to the working surface has important effects on the migration and distribution of H_2_S. Figures 9 and 10 show the distributions of H_2_S concentration in the *yz* and *xz* sections, respectively. Figures 11 and 12 show the isograms of H_2_S distribution at *z* = 2.4 in the center plane of the intercepted duct and *x* = 28 near the TBM head, respectively.

When the distance from the air duct was 3 m, because the air flow did not form a real eddy in the corner, the amount of H_2_S carried by the jet was not large, and most of the H_2_S gathered on the return air side and accumulated in the downward corner under the action of gravity. In the passive diffusion stage, most of the high-concentration H_2_S in the lower corner carried by the air flow returned to the roof of the tunnel and adhered to the return air wall. A small part of the deflecting jet diffused toward the alley wall on the side of the duct. The migration and diffusion process of H_2_S can be summarized as follows: heading > the return air corner > the return air wall outflow. When the distance from the wind duct increased from 3 to 5 m, the eddy strength decreased, the region expanded, and the jet’s enrolling air volume and rotating air volume increased; thus, the H_2_S gas emitted from the header was constantly brought into the vortex area, and most of the H_2_S was collected in the vortex center area (approximately 3.5 m away from the working face in the middle and lower part of the tunnel). The remaining H_2_S diffused toward the operating surface of the tunnel wall and the tunnel side wall with the dense eddy boundary return air. Therefore, for *L* = 5 m, the migration and diffusion process of H_2_S can be summarized as follows: header—enrichment at the front of the header fuselage—reflux through the upper part of the header. When the distance from the duct increased further to 7 m, due to the small wind speed when the jet reached the end of the working face and deflected along with the expansion of the area with small vortex strength, the volume of enrolling air and rotating air of the jet also increased significantly. The slope still converged toward the central area of the vortex when the distance from the duct was *L* = 5 m, but the intensity of this phenomenon was significantly reduced. Most of the H_2_S flowed out of the working face and dispersed in the tunnel space. Therefore, for *L* = 7 m, the migration and diffusion process of H_2_S can be summarized as follows: tunneling head—vortex center area—tunnel space dispersion reflux.

Based on the above analysis, the closer the outlet position of the air duct is to the end of the working face, the lower the concentration of H_2_S in the vortex region at the corner. The H_2_S concentration first increased and then decreased as the angle of the airflow deflecting jet increased. For *L* = 5 m, a large amount of H_2_S gas in the corner was brought into the vortex region, leading to H_2_S enrichment near the working face. For *L* = 7 m, the H_2_S gas coming from the head was rapidly dispersed in the entire tunnel space, which is not conducive to its control and treatment. For *L* = 3 m, a high concentration of H_2_S flowed back along the wall of the return air lane (diagonally upward), which is conducive to centralized pumping to improve the working environment. The suction device can be set in the middle of the tunnel wall on the return air side to centrally discharge H_2_S and dust that flow back with the air flow.

### Effect of duct diameter on the distribution of H_2_S concentration

**Figure 13:**
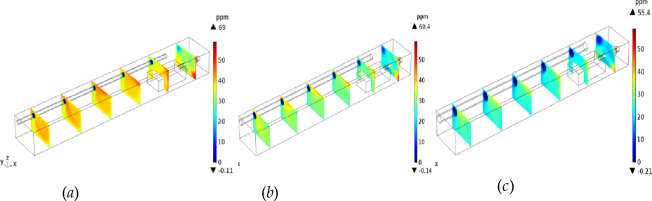
Distributions of H_2_S concentration under different duct diameters.

**Figure 14:**
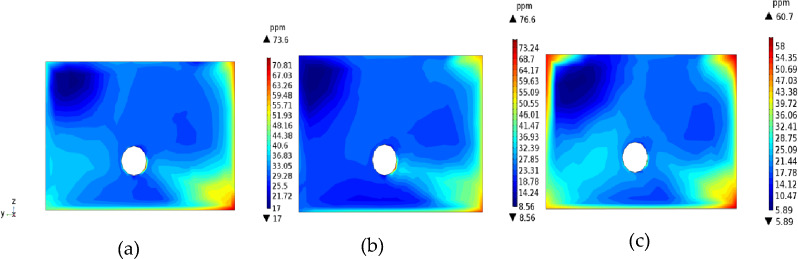
Contour maps of H_2_S concentration in the 2-m *YZ* section from the head 14.

The effect of duct diameter on the H_2_S concentration distribution was studied using the three duct radius values of *D* = 0.6, 0.8, and 1.0 m. The outlet of the duct was 3 m away from the working face, the supply air speed was 10 m/s, and the supply air temperature was 20 °C. The simulation results are shown in Figs. 13 and 14.

Among the tested values, the duct diameter of 0.4 m was most conducive to the discharge of H_2_S. When the diameter of the duct was too small (*D* = 0.6 m), it was difficult to concentrate the H_2_S in the working face and discharge it to the return air side due to the small air volume. In particular, when the tunnel header cuts the coal in the middle and lower parts of the tunnel, the H_2_S emitted from the head of the duct side is less affected by the air flow than when it does not. The harmful gas released from coal breaking cannot be sucked away and will be affected by the return air flow to the operating surface and the outside of the tunnel, which is not conducive to protecting workers from concentrated H_2_S emissions. When the duct diameter was too large (*D* = 1 m), the air volume was too large, and the wind speed near the working face was significantly higher compared with that for *D* = 0.6 and 0.8 m, causing the enfranchisement volume in the jet process to expand. In addition, due to the high return wind speed, part of the H_2_S returning from the lower corner was brought back to the wall of the air duct near the driver of the tunnel under the influence of the offset return air flow. The stagnant area at the end of the air flow was within this range, and the flow field was relatively stable. H_2_S was dispersed in the working area under the action of gravity and continued to spread outward under the influence of the tunnel air flow. This process is also not conducive to protecting the safety of operators and the centralized emission of H_2_S. As shown in Fig. 13b, for *D* = 0.8 m, the jet’s enrolling air volume was moderate. Within the effective range of the airflow, the airflow carried most of the H_2_S emitted from the head toward the return air side. The H_2_S then diffused outward along the return air side through the right side and upper side of the TBM. Therefore, when the duct diameter was 0.8 m, the H_2_S concentration near the TBM driver at the inlet air side was conducive to H_2_S removal. Most of the H_2_S gas emitted from the working face was discharged along the return air side of the working face, which is conducive to the centralized emission and treatment of H_2_S.

## Field test of H_2_S distribution

**Figure 15:**
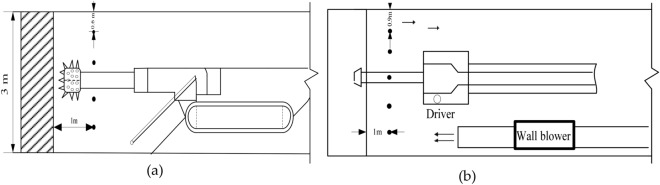
Layout of H_2_S measuring points on the excavation working face. (**a**) Arrangement of measuring points along the height direction of the top and bottom plates. (**b**) Arrangement of measuring points along the horizontal direction of the left and right sides.

To study the distribution of H_2_S concentration at the bottom, middle, and top of the boring machine, a portable CD4-type H_2_S instrument was used to analyze the distribution of H_2_S near the excavation working face. Figure 15 shows the arrangement of H_2_S measuring points on the excavation working face of the Baozigou mine. Considering the site production conditions and research needs, on the same section of tunnel near the excavation working face, a total of four measuring points were arranged along the height direction of the roof and floor, and a total of five measuring points were arranged along the left side of the tunnel to the right side of the horizontal direction. Each measuring point was 1 m away from the coal wall of the excavation working face.

### H_2_S distribution along the height direction

**Figure 16:**
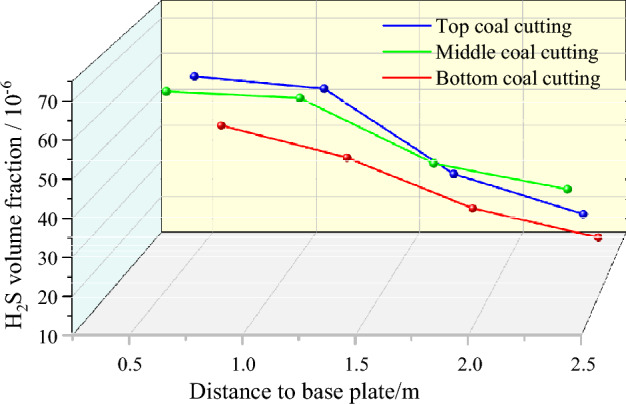
H_2_S distributions in the height direction of the roof and floor along the same section of tunnel.

As shown in Fig. 16, in the process of coal cutting by TBM, due to the layering caused by H_2_S being heavier than air, the H_2_S concentration gradually decreased moving from the tunnel floor to the roof. When TBM was used to cut the top coal, the volume fractions of H_2_S measured at 0.6, 1.2, 1.8, and 2.4 m from the tunnel floor were respectively 68.2 × 10^−6^, 65.6 × 10^−6^, 48.9 × 10^−6^, and 42.9 × 10^−6^. Due to the collapse of the coal wall and the crushing action of coal stone during coal cutting, the concentration of H_2_S emitted by the TBM when cutting the top and middle coal seams was higher than that when cutting the bottom coal. At the measuring point 1.2 m away from the tunnel floor, the volume fractions of H_2_S measured when cutting the top and middle coal seams were 65.6 × 10^−6^ and 61.2 × 10^−6^, respectively, which were 2.5*–*3 times greater than that measured when cutting the bottom coal seam. Therefore, when controlling H_2_S hazards during coal cutting by TBM, we should focus on the middle and top coal seams while also considering the bottom coal seam.

### Distribution of H_2_S in the horizontal direction

**Figure 17:**
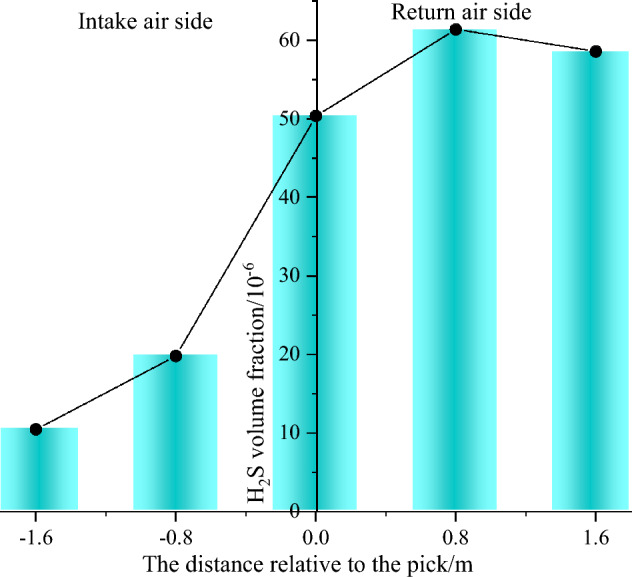
H_2_S distributions in the horizontal direction of two sides along the same section of tunnel.

The distribution of H_2_S in the horizontal direction of two sides of the same section of tunnel near the working face was evaluated for the TBM cutting of the middle coal seam. The breathing zone (*z* = 1.55) plane of the worker was taken as the reference plane, and the distances between the pick head and its left and right sides were 0.8 and 1.6 m, respectively. Figure 17 shows the distribution of H_2_S in the horizontal direction. Under the influence of a U-type ventilation system on the excavation working face, the H_2_S concentration gradually increased along the left side of the tunnel from the head of the pick to the right side of the return air side. The volume fraction of H_2_S reached 61.2 × 10^−6^ and 59.4 × 10^−6^ when the horizontal distances from the pick head on the return air side were 0.8 and 1.6 m, respectively; these volume fractions were respectively 3 and 5.36 times those at the same position on the inlet air side. Thus, when treating H_2_S hazards near the cutting head of the excavation working face, it is necessary to focus on the area from the cutting head to the return air side.

## Conclusions

This research examined the source of H_2_S emissions, the features of H_2_S migration, and the distribution of H_2_S concentrations in the 10103 excavation working face of the Baozigou coal mine by numerical simulations and field measurements. The main conclusions are as follows:

1. BSR and TSR are the principal sources of H_2_S in the Baozigou coal mine.
2. According to the air flow characteristics of the excavation working face, the diffusion process of H_2_S can be divided into the gravity diffusion stage, passive diffusion stage, and uniform diffusion stage after gushing from the 10103 excavation working face of the Baozigou coal mine.
3. Elevated wind speed facilitate the release of H_2_S gas; on the other hand, excessive wind speed is detrimental to the concentrated collection and assimilation of H_2_S. It was found that 10 m/s was the optimal wind speed at the wind duct’s exit. The H_2_S concentration in the vortex area at the corner is lower the closer the air duct's outlet location is to the working face's end. The H_2_S concentration increases and subsequently decreases with an increase in the air flow deflection jet's angle. When the diameter of the duct is too small, the H_2_S released by the hard-to-break coal cannot be sucked away. When the diameter of the duct is too large, the suction volume in the jet process will increase due to the high return windspeed, and a portion of the H_2_S gas will return to the side wall of the duct near the driver of the boring machine.
4. Measurements of H_2_S concentration during coal cutting at the bottom, middle, and top coal seams indicate that the wind at the outlet of the wind duct, the installation position of the wind duct, and the diameter of the wind duct can be adjusted to reduce the eddy current formed at the intersection of the inlet air and the return air on the excavating face.

## Discussion

The results of the test showed that by adjusting the wind speed, position and diameter of the windmill to reduce the turbulence generated at the intersection between the incoming and returning winds of the work surface, H_2_S emissions were not sufficient to be reduced to less than the national standard concentration, and still had an impact on staff. It is recommended to use other means to further reduce the H_2_S concentration based on actual working conditions. This is also what I need to conduct further research on next, striving to control H_2_S and reasonably improve the working environment of the staff.

## Data Availability

All data generated or analysed during this study are included in this published article.
